# Referred pain: characteristics, possible mechanisms, and clinical management

**DOI:** 10.3389/fneur.2023.1104817

**Published:** 2023-06-28

**Authors:** Qianjun Jin, Yuxin Chang, Chenmiao Lu, Lunhao Chen, Yue Wang

**Affiliations:** Department of Orthopedic Surgery, The First Affiliated Hospital, Zhejiang University School of Medicine, Hangzhou, China

**Keywords:** referred pain, somatic, spinal, neuropathic pain, central sensitization

## Abstract

**Purpose of this review:**

Referred pain is a common but less understood symptom that originates from somatic tissues. A comprehensive recognition of referred pain is important for clinicians when dealing with it. The purpose of this study is to summarize the current understanding of referred pain, including its pathogenesis, characteristics, diagnosis, and treatment.

**Recent findings:**

Referred pain arises not only from pathologies primarily involving local tissue but also from lesions in distant structures. Central sensitization of convergent neurons and peripheral reflexes of dichotomizing afferent fibers are two theories proposed to explain the pathological mechanism of referred pain. Because syndromes related to referred pain of different origins overlap each other, it is challenging to define referred pain and identify its originating lesions. Although various approaches have been used in the diagnosis and treatment of referred pain, including conservative treatment, blockade, radiofrequency, and surgery, management of referred pain remains a clinical challenge.

**Summary:**

Unlike radicular pain and neuropathic pain, referred pain is a less studied area, despite being common in clinics. Referred pain can derive from various spinal structures, and blockage helps identify the primary pathology. Due to the heterogeneity of referred pain, treatment outcomes remain uncertain. Further studies are needed to improve our understanding of referred pain.

## Introduction

In the field of somatic pain, most clinicians are familiar with radicular pain and neuropathic pain, but little is known about referred pain. Referred pain occurs in an area far from the primary lesion (1, 2) and is sometimes associated with secondary hyperalgesia and trophic changes in the referred areas (3). Although clinically common, the nature of referred pain remains an enigma (4–6). In most cases, pain in the dermatome regions, which are innervated by specific peripheral nerves, is simply regarded as radicular pain or neuropathic pain (1, 7). Referring to this phenomenon, the International Association for the Study of Pain (IASP), citing the classic treatise on pain, stated long ago in 2011 that “Pain in the lower limb should be described specifically as either referred pain or radicular pain” [*sic*] (2). In addition to the lower limbs, the diagnosis of pain in other somatic areas should also follow this guideline. Some researchers have even suggested that if the nature of pain is not clear, the diagnosis should not be jumped to Vulfsons et al. (7).

A better understanding of referred pain may help physicians in clinical diagnosis and decision-making, thus improving clinical outcomes. To this end, this review characterizes referred pain in various conditions and discusses possible pathological mechanisms and therapeutic measures based on the contributions of previous studies.

## Definition and epidemiology of referred pain

Somatic pain arises not only from pathologies primarily involving local tissues, but also from dysfunction in distant tissues, including spinal, neuromuscular, and other somatic structures (8, 9). The term “referred pain” has been documented to describe pain spreading to the somatic regions far from the site of noxious stimulation (10), which is not caused by nerve root stimulation.

Referred pain can be caused by autogenous dysfunction or triggered by external stimuli. For example, referred pain can even be stimulated by hypertonic saline in healthy adults (11, 12). However, referred pain is not directly caused by mechanical or inflammatory stimulation of the nerves or nerve roots (1), nor is it caused by neuromas or lesions of the peripheral nerves or central nervous system (13). On the contrary, referred pain has even been induced by stimulating adjacent structures in patients with denervated or missing limbs in special cases of spinal cord injury (14), plexus avulsion (15, 16), and amputation (13, 17). In another typical example, referred pain has been triggered in areas of neuropathic pain by scraping the earlobe in patients with postherpetic neuralgia (18).

It is clear that referred pain is common and variable. Spinal referred pain, for example, reportedly occurs in 17%–84% of patients with low back pain (19, 20). While referred pain has been a focus of research for a long time, multicenter studies are lacking, and the incidence of referred pain in the general population largely remains unknown.

## Possible pathophysiology of referred pain

**Figure 1:**
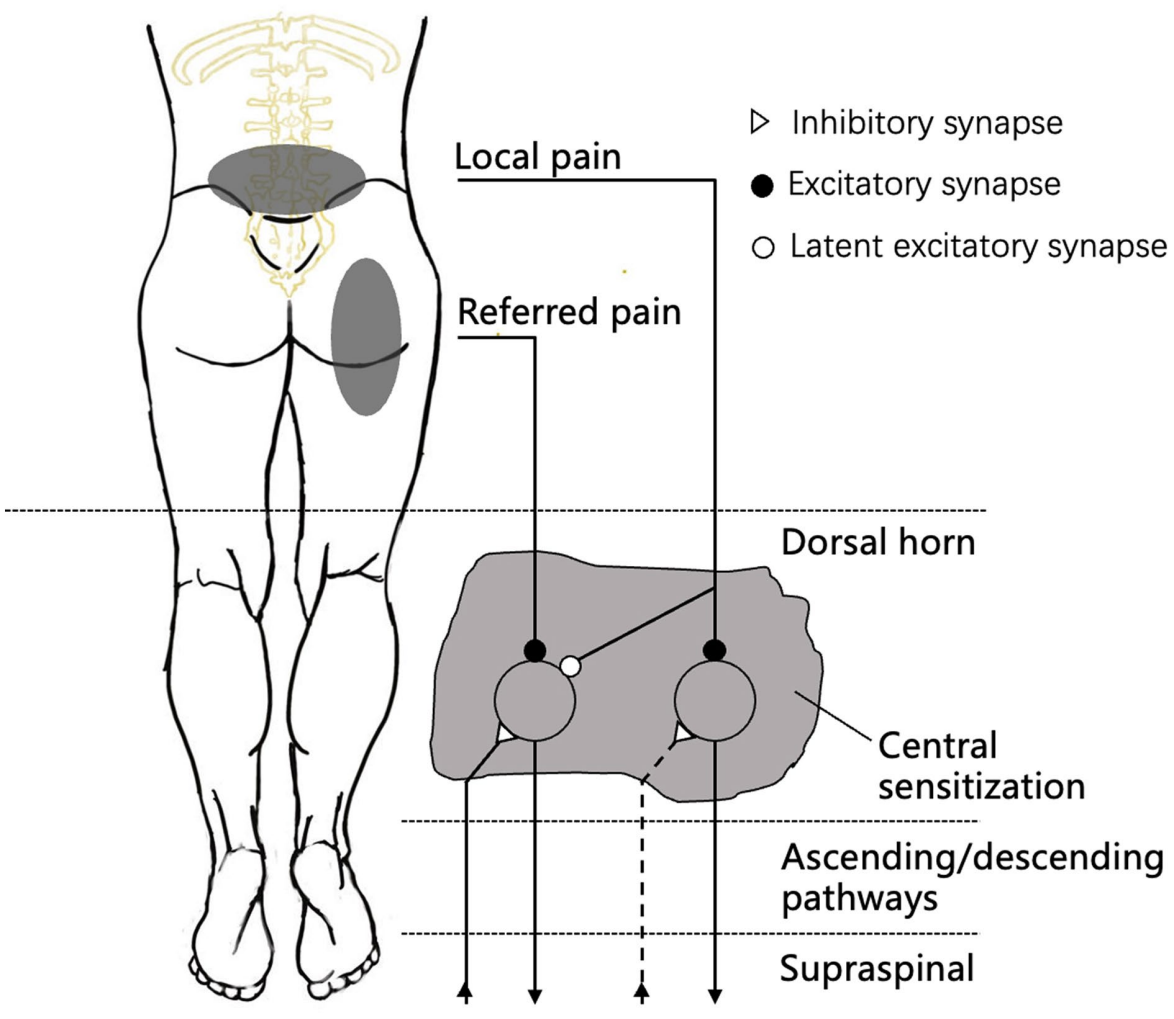
The neurophysiological schematic model of “central sensitization” in humans. Local pain will excite the pathway mediating pain upward to the dorsal horn, while a sensitization process is initiated at the same time. The release of sensitizing substances will cause the opening of latent synaptic connections to the neurons mediating pain from the referred area. Both the direct excitation of the involved neurons and the facilitation of afferent input in the referred area will result in the perception of referred pain (5).

The cortical reorganization theory has long been proposed to explain the pathophysiology of referred pain (21). Contrary to the cortical reorganization theory, however, referred pain has also been found in areas with segregated cortical activation (22). In general, most researchers accept convergence projection theory. In this theory, the convergence of nociceptive afferents on second-order neurons in the spinal cord leads to the occurrence of pain in different somatic areas, like “crossed telephone lines” (9, 10, 23, 24) (Figure 1). While the convergence of sensory afferents at the subcortical level has been confirmed in an animal study (25), referred pain can be considered the central sensitization of convergent neurons. A classic example is the convergence of C1–C3 spinal nerves, where trigeminal input results in migraine and cervicogenic headache (26).

**Figure 2:**
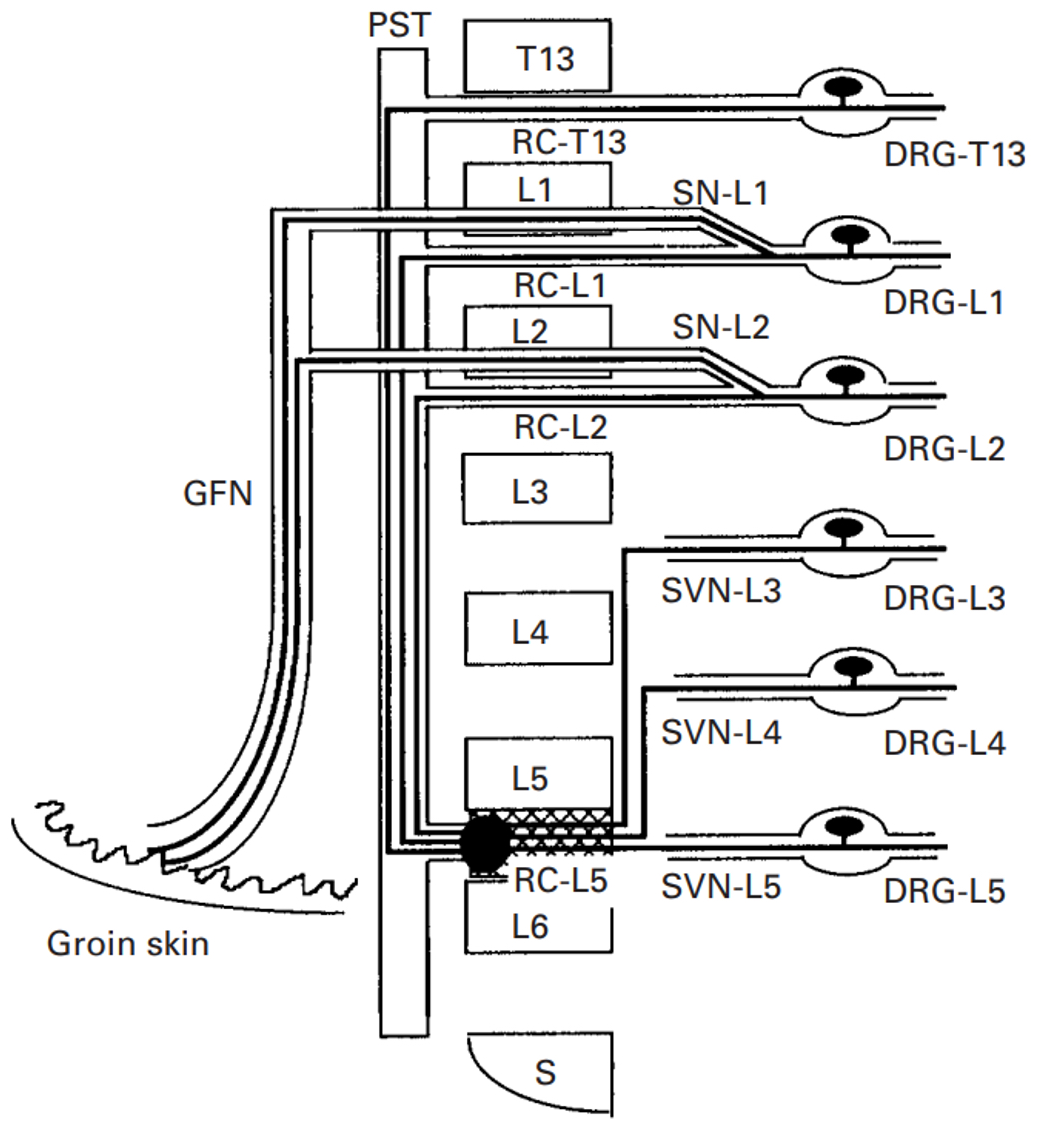
The neurophysiological schematic model of “dichotomizing axons” in rats. There are dichotomizing axons that project to the ventral portion of the L5–L6 disc and the groin skin during the sensory pathways from the ventral portion of the L5–L6 disc and neurons in the L1 and L2 DRG. GFN, genitofemoral nerve; PST, paravertebral sympathetic trunk; RC, ramus communicans; SN, spinal nerve; SVN, sinuvertebral nerve (30).

In another theory, it has been proposed that there are dichotomizing afferent fibers that ramify and distribute to regions of primary dysfunction and referred areas (27–30). While primary lesions stimulate afferent fibers in deep regions, these afferent fibers trigger the activation of a reflex arc toward the muscle via somatic efferent fibers. This theory can be summarized as the pathomechanism underlying physiological reflexes (8). Using double-labeling with fluorescent tracers, dichotomizing axons between the lumbar intervertebral disc and the groin region have been reported (30), supporting this theory (Figure 2). Another example is visceral referred pain, which is regarded as a pathological combination of nociceptive processing pathways for visceral and somatic sensory afferent neurons. As such, visceral referred pain is also called a “viscerosensory reflex” (9, 31).

## Referred areas for lesions in various spinal structures

Reported referred pain can originate from almost all spinal structures (5, 31). Among them, the intervertebral disc (31), facet joint (32, 33), and sacroiliac joint (34) are the most common structures involved in spinal referred pain. Referred pain is usually felt in some designated dermatome areas in patients with various spinal disorders, including spinal injuries, disc degeneration, spinal canal stenosis, and lumbar spondylolisthesis (34–36). For example, the pain usually occurs in the distant lumbosacral regions in patients with traumatic or osteoporotic vertebral fractures (37, 38), suggesting that pain may be referred outward and downward from the fracture site according to some predictable patterns. Kellgren et al. first reported that spinal referred pain may be distributed in a segmental pattern (39, 40). However, it is difficult to “map” referred areas because referred pain of different origins often overlaps (12). Moreover, referred pain does not follow dermatomes or patterns of nerve root distribution (11, 41).

While referred pain in the chest, shoulder, and upper limb usually originates from the cervical and thoracic spine (11, 42, 43), referred pain related to lumbar spine diseases tends to spread to the back, abdomen (24), buttocks (12, 31, 44, 45), groin (12, 46, 47), thigh (12, 34), and even the distal limb below the knee (48). Among them, the buttocks and leg are the most common sites of lumbar referred pain (12, 28, 31, 34, 49). Moreover, the distribution patterns for lumbar referred pain vary according to anatomical structures. For example, patients with disc degeneration may feel pain at the dermatome region innervated by the corresponding dorsal root ganglion (DRG) neurons (1, 19, 31, 48, 50), with greater intensity of referred pain being associated with more severe disc degeneration (31, 48).

Referred pain originating from the facet joints is also segmentally distributed, as it may be related to segmental DRGs. For cervical facet joints, the neck and shoulder are the most commonly involved sites of referred pain (42, 51). Referred pain in the back and iliac crest usually originates from the thoracic facet joints (43). For lumbar facet joints, pain may be referred to as the region between the hip and thigh. It has been reported that pathology of the superior facet joints typically induces referred pain to the flank, hip, and lateral thigh regions, while the inferior facet joints usually refer to the posterior thigh (12, 33, 52).

Referred pain from the sacroiliac joint mainly distributes to the lower back and buttocks, in addition to the thigh, groin, leg, and even foot (31, 53, 54). Nevertheless, there is considerable overlap in referral areas, although the pain has been irradiated from various spinal elements (54). A clear map specifying the relationship between anatomical structures and referred regions is currently lacking.

## Differentiation of referred pain from radicular pain

Referred pain usually occurs after local pain has persisted for a certain period (5, 55). Typically, referred pain is described as dull, aching, gnawing, annoying, drilling, or pressing (1, 55). Sometimes, referred pain is associated with secondary hyperalgesia and trophic changes (3). Once present, referred pain tends to become fixed in a particular region, depending on the referral pattern (1, 55). Unlike the explicit pathophysiology of radicular pain, it is difficult to locate the exact original site or boundaries of the affected area for referred pain as the convergence of afferent neurons is rather complicated (24, 34, 40, 44). However, referred pain can be identified in a wide area with an identified center or core, although its boundary is difficult to define (11). Although a number of studies have described the segmental patterns of spinal referred pain, these patterns are not consistent among patients and dysfunctions (11, 31, 34, 43, 44). Importantly, referred pain is not dermatomally distributed, which is markedly different from radicular pain (11, 41).

Neuropathic pain is a symptom and also a consequence of various diseases affecting the somatosensory nervous system (13, 56). Typically, neuropathic pain is described as allodynia and hyperalgesia and is usually distributed along the dermatome of peripheral nerve-innervated areas (13, 24). Mechanistically, radicular pain is evoked by ectopic discharges from affected DRGs or nerves. For example, herniated disc tissues compress the spinal root and induce pain traveling along the lower limb, which may be largely resolved after the removal of the herniated disc tissue (57, 58). In many cases, radicular pain is associated with neurological deficits such as decreased muscle strength, weakened reflexes, and specific numbness in the related dermatome (59, 60). The neurophysiological relationship between radicular pain and the affected nerves has been clarified in preclinical animal studies (61–63). The distinguishing features of referred pain and radicular pain are listed in Table 1. However, how to precisely identify referred pain and distinguish it from radicular pain needs further investigation.

**Table 1:** Differential features of referred pain and radicular pain.

Feature	Referred pain	Radicular pain
Quality	Dull, aching, gnawing, annoying, drilling, pressing	Shooting, lancinating, shocking, electric
Concomitant symptom	Secondary hyperalgesia trophic changes	Numbness, decreased muscle strength, weakened reflexes
Distribution pattern	A wide area with a boundary that is difficult to define, but with an identified center or core	Travels along the length of the lower limb in a band no more than 2–3 inches in width

## Diagnostic measures for referred pain

As mentioned above, referred pain can originate from various spinal elements, and there is significant overlap in referred areas and distribution patterns. It is thus difficult to locate the exact source of referred pain (24). There are two methods that can help diagnose referred pain: (1) to induce a similar pain pattern using different types of stimuli; (2) to relieve the pain with a local block (5, 31, 55, 64).

In many cases, radiographic imaging, such as CT, MR, and myelography, is not able to identify the exact source of referred pain. As early as 1939, Kellgren et al. first developed an experimental model and found that referred pain could be triggered by injection of hypertonic saline solution (39, 40, 65), as verified in investigating cases of spontaneous pain of unclear etiology (6). Extending this theory, various percutaneous procedures, namely provocative lumbar discography (66–70), facet joint injections (32), blocks (33, 52, 70), intra-articular sacroiliac joint injections (31, 53, 54), and epidural injections (70) have been developed to reproduce or block referred pain and to identify its exact source. In an inflammatory pain model, capsaicin has been used for functional spinal and supraspinal MR imaging, which has been reported to identify the origin of referred pain (18).

## A multidisciplinary approach to the treatment of referred pain

Multimodal treatments for referred pain include pharmacotherapy, physical therapy, regular exercise, and psychotherapy (52). Invasive procedures are reserved for those with a confirmed source of somatic referred pain. Invasive therapies, such as local blockage and radiofrequency, are commonly used in treating spinal referred pain. In an early Lancet report, referred pain triggered by injecting hypertonic saline solution could be successfully treated with a local block (6). Later, Hockaday et al. reported that referred pain could be abolished by a local block but was less consistently reduced by blocking the referred areas (11). For instance, intra-articular injections of corticosteroids have been used to treat facetogenic (52, 71) and sacroiliac-referred pain (72). Furthermore, disco block, inspired by discography, was once regarded as a useful approach to relieving discogenic referred pain (50, 73). Shealy et al. first described the method of applying radiofrequency to the facet joints in 1975 (74). Radiofrequency has since been acknowledged as the “gold standard” for the treatment of facetogenic and sacroiliac-referred pain. Thermal intradiscal procedures, such as intradiscal electrothermal annuloplasty and biacuplasty, have also been used to effectively treat discogenic low back pain and referred leg pain (20, 31, 75).

When treating referred pain, non-invasive treatments are usually optional (76). There are some exceptions. For example, discogenic groin pain can be significantly improved after lumbar disc surgery (50). Percutaneous vertebroplasty, or kyphoplasty, is effective in relieving lumbosacral referred pain following osteoporotic vertebral compression fractures (38). It has also been reported that patients with back pain associated with referred inguinal or leg pain had better clinical outcomes after spine surgery than those without (77), suggesting that surgical therapy for the primary conditions may be an effective approach to controlling the secondary referred pain.

Although various interventions have been reported to be effective in the treatment of referred pain, randomized controlled trials are needed to clarify the efficacy of conservative treatments, blockage, radiofrequency, and surgery. In addition, clinical guidelines and expert consensus on the management of referred pain are currently lacking.

## Conclusion

In this mini-review, patterns, and characteristics of referred pain from somatic structures are discussed. The similarity between referred pain and peripheral neuropathic pain makes it difficult to approach the diagnosis of referred pain. The main hypotheses for the pathophysiological mechanism of referred pain include central sensitization and peripheral reflex, which may jointly explain the development of referred pain. Convergence of sensory afferents at the subcortical level and dichotomizing afferents have also been proposed in scientific research. The overlap of referred areas from different somatic structures makes it difficult to locate the primary origins of referred pain. In clinical practice, local blockage and radiofrequency are commonly used interventions in the diagnosis and treatment of referred pain, although there is a considerably high false-positive rate for both. Given the complex pathologies, changeable manifestations, and inconsistent distribution patterns, the treatment of referred pain remains a clinical challenge.

## Author contributions

QJ and YW carried out the project design and literature summary. All authors contributed to the article and approved the submitted version.

## Funding

This work was supported by the National Natural Science Foundation of China (grant no. 82201358) and the Medicine and Health Science and Technology Plan in Zhejiang Province (grant no. 2023RC019).

## Conflict of interest

The authors declare that the research was conducted in the absence of any commercial or financial relationships that could be construed as a potential conflict of interest.

## Publisher’s note

All claims expressed in this article are solely those of the authors and do not necessarily represent those of their affiliated organizations, or those of the publisher, the editors and the reviewers. Any product that may be evaluated in this article, or claim that may be made by its manufacturer, is not guaranteed or endorsed by the publisher.
